# The position and morphometry of the fovea capitis femoris in computed tomography of the hip

**DOI:** 10.1007/s00276-018-2097-y

**Published:** 2018-08-31

**Authors:** Marcin Ceynowa, Marek Rocławski, Rafał Pankowski, Tomasz Mazurek

**Affiliations:** Department of Orthopedic Surgery, Medical University of Gdańsk, ul. Nowe Ogrody 1-6, 80-803 Gdańsk, Poland

**Keywords:** Femur, Hip, Fovea, Computed tomography

## Abstract

**Purpose:**

The position of the fovea of the femoral head is usually considered to be inferior or inferoposterior, despite the fact that few detailed anatomical studies have been performed. This study was performed to assess the position of the fovea in computed tomography and its correlation with standard radiographic measures of the proximal femur.

**Methods:**

Computed tomography scans of the hip of 107 patients (54 women and 53 men) were evaluated. The semi-coronal and transverse views were used to assess the femoral neck–shaft angle and the neck version, as well as the size and position of the fovea in relation with the femoral neck axis and the size of the head.

**Results:**

The fovea was always located inferior to the neck axis in the semi-coronal plane. In the transverse plane, the fovea was always slightly posterior to the femoral neck axis, as approximately ¾ of its diameter was posterior to the axis. The position was unrelated to the neck–shaft axis and the neck–trochanter minor angle. There were no differences in the position between men and women; however, in women, the fovea is slightly larger than in men when related to the femoral head size.

**Conclusion:**

The femoral neck axis in the transverse plane always crosses the anterior aspect of the fovea. Its position is unrelated to the angular geometry of the proximal femur, but related to the femoral head size. It is found to be relatively larger in women.

## Introduction

The position of the fovea of the femoral head has been determined in anatomy books seemingly once and for all. It is usually described as positioned inferiorly on the femoral head, with no regard to its anteroposterior direction [1]. Although some authors cite its slightly posterior position from other sources [2–6], few detailed anatomical studies have been performed [1, 7, 8].

There are a few specific circumstances when the knowledge of the position of the fovea of the femoral head may be important. It is increasingly used as one of the measures in evaluating radiographs of the developmental dysplasia of the hip, serves as a landmark in hip arthroscopy, and is used in anthropological practice [4, 9, 10]. Assessment of fovea position in radiography may help to determine the rotational position of the femoral head in femoral neck fracture fixation [11]. The knowledge of the exact position of the fovea is also crucial in ligamentum capitis femoris reconstruction [2, 7, 12, 13].

This study was performed to assess the position of the fovea and its correlation with standard radiographic measures of the proximal femur.

## Materials and methods

Random computed tomography (CT) scans of the hip and pelvis of 107 patients from the database of the Copernicus Hospital in Gdańsk were evaluated. Mean age was 43.42 years (SD 11.7, range 18–60). There were 54 women and 53 men. Women mean age was 45.28 (SD 11.31), and mean age of men was 41.3 (SD 11.9). The differences in age were statistically insignificant (*p* = 0.09).

Patients younger than 18 years were excluded because of a potential risk of incomplete maturation of bones, and older than 60 years were excluded a priori patients with higher risk of osteoarthritis or severe osteoporosis. CT was performed in course of their clinical diagnostics and management for pathologies unrelated to hip and pelvis pathology (angiography, abdominal soft-tissue pathologies, and polytrauma assessment) in years 2016–2017. Patients with obvious hip pathology, such as fractures, osteoarthritis (Tonnies grade 2 or more), tumors, osteonecrosis, dysplasia, or other bony deformation, were excluded from the study. Only the scans that covered both iliac wings proximally and at least 2 cm distal to the lesser trochanters distally were taken into consideration. Both hips in one patient were evaluated for comparison. CT was assessed in radiological bone window. The slices thickness was 1 mm. 3D coronal and transverse reconstructions were used.

Initially, a semi-coronal plane was directed through the longitudinal axis of the femoral neck using the transverse plane on a 3D reconstruction. The transverse window was then scrolled down to make sure that the semi-coronal plane passes through the femur to the level below the lesser trochanter. Since the femur bends slightly with the convex side anteriorly, the plane should pass slightly posteriorly in the femoral shaft below the lesser trochanter. Then, the exact perpendicular windows (semi-coronal and transverse) were used for further evaluation.

**Fig. 1:**
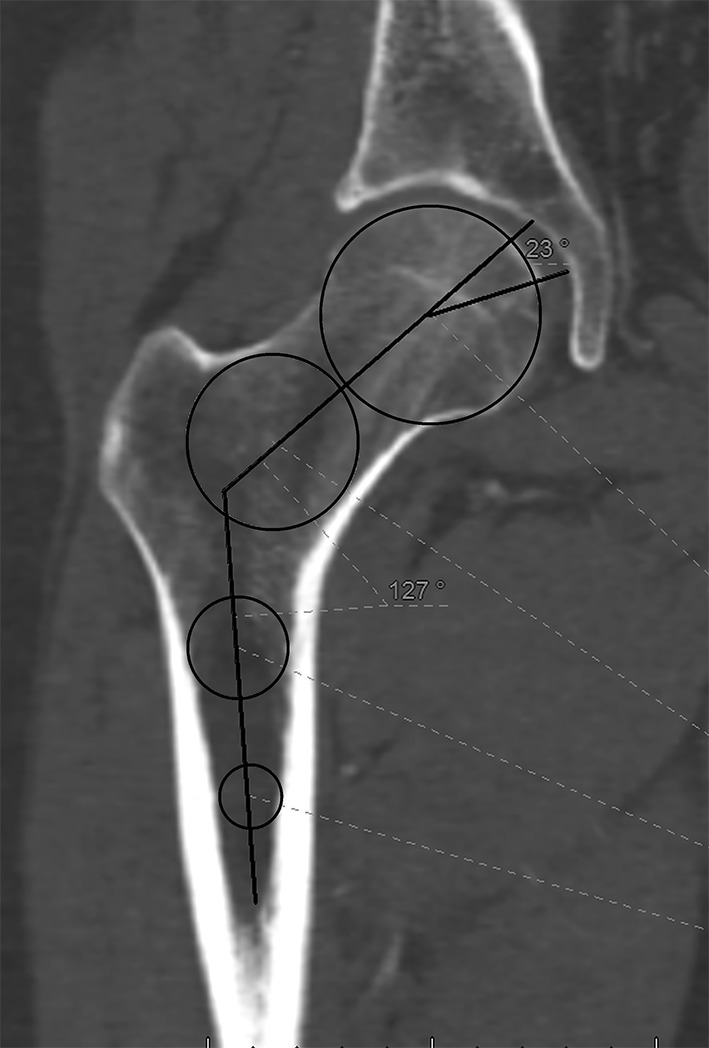
Semi-coronal view of the femur shows the neck–shaft angle and the fovea angle

The semi-coronal plane, that corresponded to the exact anteroposterior view of the femur, was used to assess the neck–shaft angle, the size of the femoral head, the size of the fovea, and the position of the fovea in relation with the neck axis (fovea angle), as shown in Fig. 1. The femoral neck longitudinal axis was drawn in both planes, using the slices, where the femoral head was at its greatest diameter. The midpoint between the cortices in the femoral neck and the middle of the femoral head served as reference points for determining the axis of the neck in the semi-coronal plane. Centroids were used to exactly determine the midpoints of the femoral head and between cortices (Fig. 1). The axis of the femur was determined as a line passing through two points: one midway between the femur cortices approximately 2–3 cm distally to the lesser trochanter (as far distally the range of the scan and the quality of the image allowed) and one at the upper border of the lesser trochanter. The angle was measured at the intersection between those two lines. A CT scan of the whole femur was available in 13 cases, and the accuracy of this measurement was checked with relation to the shaft axis of a whole femur semi-coronal scan. It showed that the measurement of the femoral shaft axis on the shorter scans was accurate within a mean of 2.100 (range 0–3) deviation from the whole bone scan, what was considered a good approximation between both methods.

The position of the fovea in the semi-coronal plane was assessed as the angle between the femoral neck axis and a line drawn between the center of the femoral head and the upper border of the fovea.

**Fig. 2:**
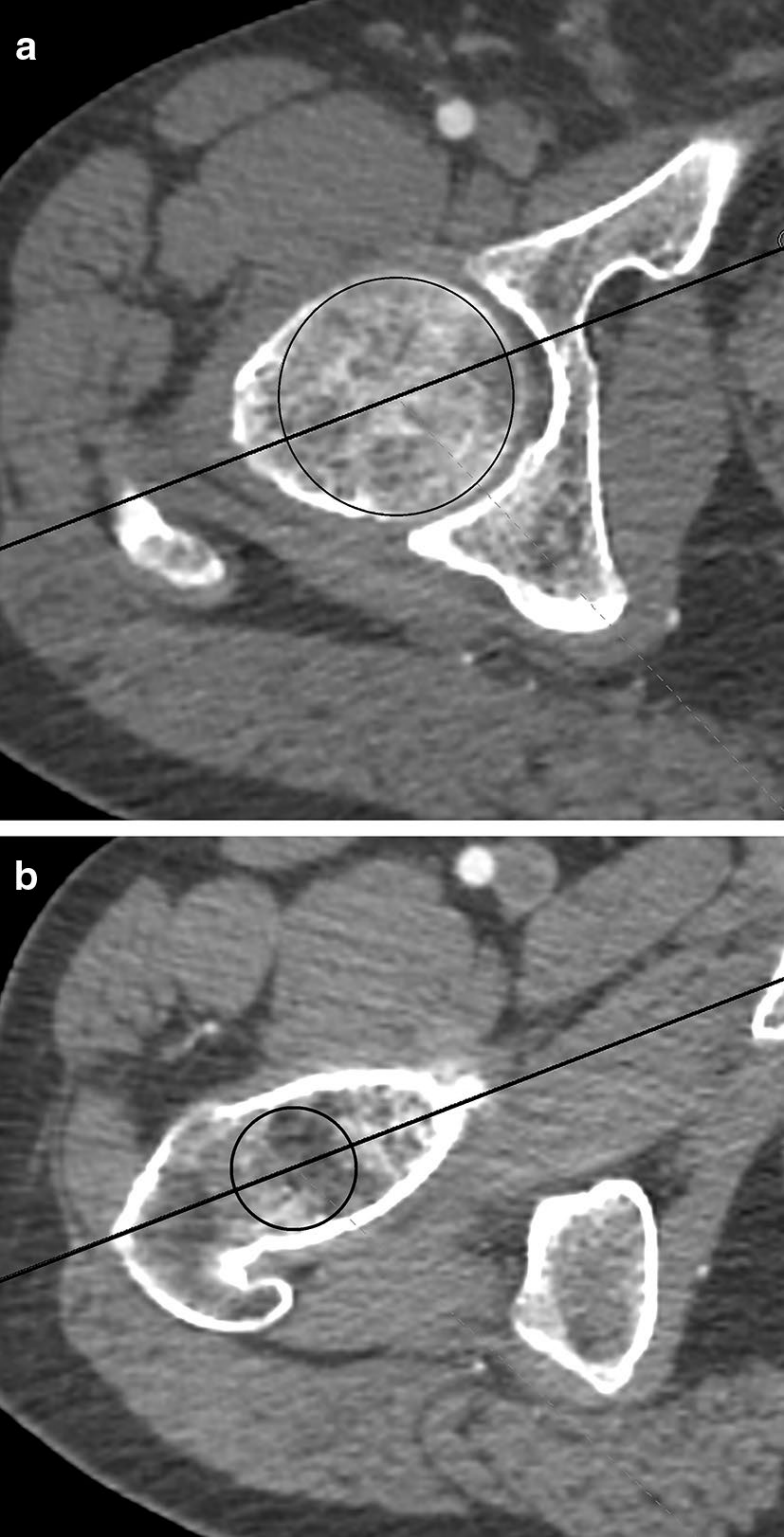
Determining the femoral neck axis in transverse view. **a** At the level of the femoral head. **b** At the level of the femoral neck basis

In the transverse plane, the femoral neck axis was measured according to Reikerals et al. [14, 15] (Fig. 2a, b). It was used to assess the diameter of the femoral head and the fovea, the position of the fovea in relation with the neck axis, and the angle between the neck axis and the axis of the lesser trochanter [16].

**Fig. 3:**
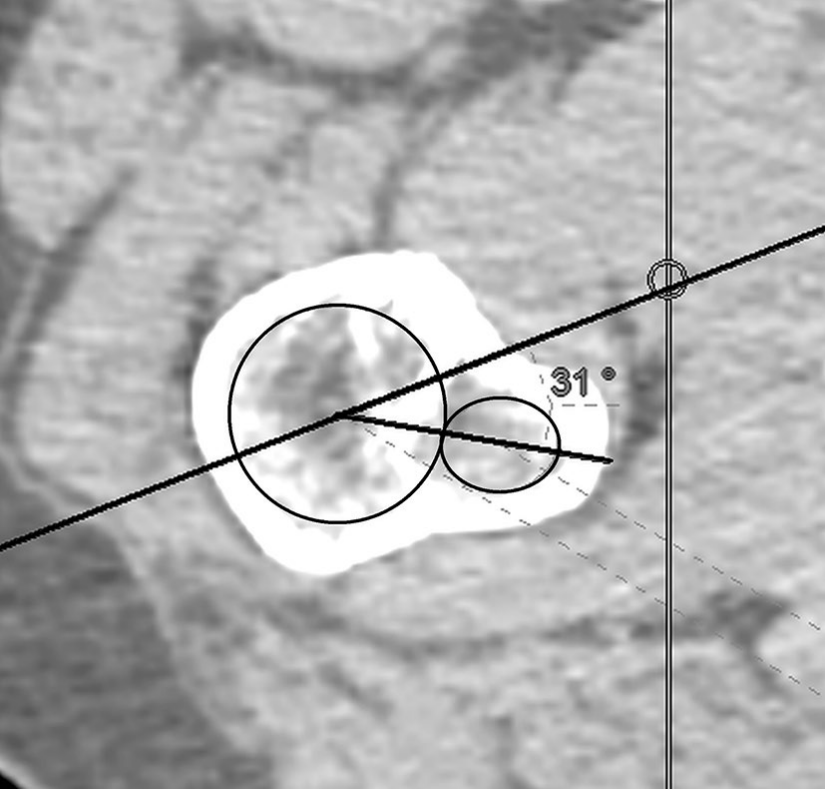
Determining the angle between the femoral neck axis and the trochanter minor in the transverse view

The relative position of the fovea in the semi-coronal plane was assessed descriptively. The relative position of the fovea in the transverse plane was measured as the distance between the axis as described above and the posterior border of the fovea (Fig. 3).

**Fig. 4:**
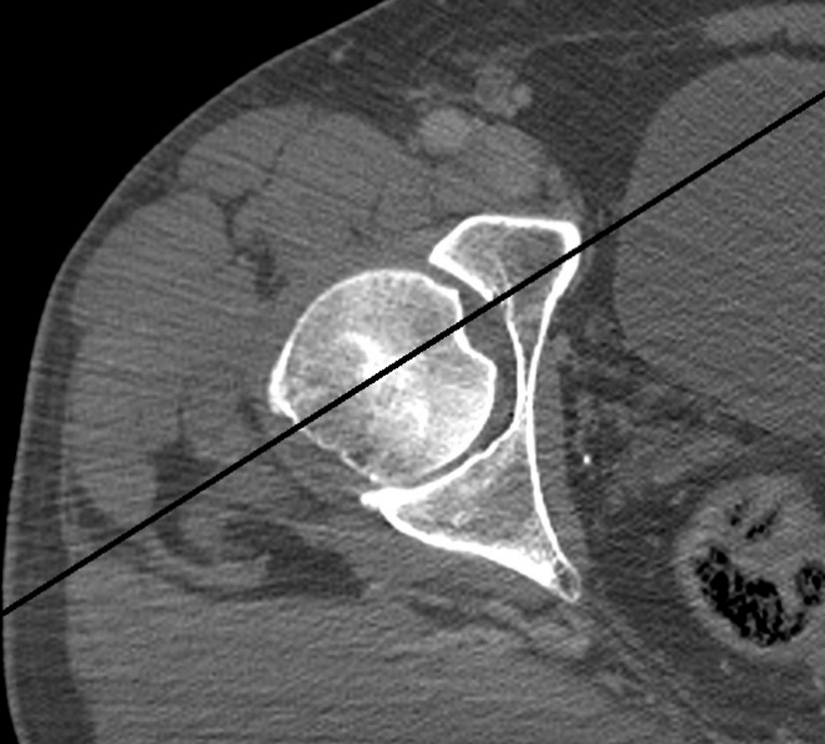
Determining the intersection between the femoral neck axis and the fovea in the transverse view

The exact point, where the femoral neck axis in the transverse plane crossed the fovea, was determined by dividing the distance between the posterior border of the fovea and the point of intersection of the axis with the femoral head cortex, by the size of the fovea. The resulting number is percentage of the fovea diameter that is located posteriorly to the femoral neck axis in the transverse plane (Fig. 4).

The femoral head diameter was calculated as a mean of the largest diameters in the semi-coronal and transverse planes. The relative size of the fovea in both planes (Acar’s index) [2] was calculated as follows: fovea diameter/femoral head diameter × 100.

The results from both hips were evaluated together (pooled), what increased sample size and, therefore, the reliability of the mathematical calculations. Since pooling may cause significant study bias (symmetry of human body, duplication of some information such as gender and age), we also calculated all the parameters using results from only one hip (left) from each subject.

Statistical analysis was performed using the Statistica ver. 13 software (Statistica, Tulsa, Oklahoma, USA). Spearman correlation coefficient was used to assess correlation between the fovea position in the transverse plane and the relative rotational position of the femoral neck axis and the lesser trochanter, as well as the correlation between fovea position in the semi-coronal view and the femoral neck–shaft angle. The Shapiro–Wilk test was used to determine data distribution, and the *t* student test was used to assess differences between male and female subjects, as well as between both hips.

## Results

The femoral neck–shaft angle, the neck–trochanter minor angle, and the relative position of the fovea to those angles are presented in Table 1. The size of the femoral head and the relative size of the fovea are presented in Table 2.

**Table 1:** Femoral angles and their relationship to the position of the fovea

	Percentage of the fovea transverse diameter posterior to the neck axis	Fovea angle	Neck–shaft angle (degrees)	Neck–trochanter minor angle (degrees)
Male				
Pooled data	72.21 (SD 14.99, range 50–100)	12.38 (SD 7.39, range 0–28)	129.19 (SD 4.03, range 119–138)	34.09 (SD 8.14, range 3–53)
Single hip data	71.76 (SD 14.84, range 50–100)	12.23 (SD 7.39, range 0–28)	129.01 (SD 4.05, range 119–136)	34.32 (SD 7.88, range 5–53)
Female				
Pooled data	77.16 (SD 15.45, range 50–100)	6.53 (SD 5.37, range 0–18)	129.5 (SD 4.72; range 118–141)	35.83 (SD 7.41, range 10–55)
Single hip data	76.66 (SD 16.0, range 50–100)	6.53 (SD 5.73, range 0–18)	129.25 (SD 4.93; range 118–141)	35.29 (SD 7.59, range 10–50)
Average				
Pooled data	74.71 (SD 15.39, range 50–100)	8.29 (SD 6.91, range 0–28)	129.35 (SD 4.38, range 118–141)	34.97 (SD 7.81, range 3–55)
Single hip data	74.23 (SD 15.56, range 50–100)	9.36 (SD 7.17, range 0–28)	129.14 (SD 4.5, range 118–141)	34.81 (SD 7.71, range 3–53)

**Table 2:** Size of the femoral head, the fovea, and the relative size of the fovea (Acar’s index) in two planes

	Fovea diameter semi-coronal (mm)	Fovea size transverse (mm)	Head diameter (mm)	Acar’s index semi-coronal	Acar’s index transverse
Male					
Pooled data	11.17 (SD 2.48, range 4–16)	13.43 (SD 2.78, range 6–20)	49.81 (SD 2.97, range 44–59)	22.25 (SD 4.57, range 8.7–30.8)	27.08 (SD 5.24, range 12–41.6)
Single hip data	11.30 (SD 2.65, range 4–16)	13.41 (SD 2.87, range 7–20)	49.87 (SD 3.08, range 44–59)	22.46 (SD 4.8, range 8.7–30.8)	26.98 (SD 5.31, range 14.28–41.6)
Female					
Pooled data	10.5 (SD 2.12, range 5–16)	12.46 (SD 2.35, range 5–17)	43.65 (SD 2.29, range 40–52)	23.83 (SD 4.59, range 12.2–34.1)	28.8 (SD 5.18, range 11.9–40.5)
Single hip data	10.74 (SD 2.18, range 6–16)	12.42 (SD 2.43, range 5–17)	43.72 (SD 2.34, range 40–52)	24.33 (SD 4.73, range 14.2–34.1)	28.64 (SD 5.38, range 11.9–49)
Average					
Pooled data	10.83 (SD 2.32, range 4–16)	12.94 (SD 2.61, range 5–20)	46.55 (SD 4.42, range 40–58)	23.05 (SD 4.69, range 8.7–34.1)	27.93 (SD 5.2, range 11.9–41.6)
Single hip data	11.01 (SD 2.43, range 4–16)	12.91 (SD 2.69, range 5–20)	46.77 (SD 4.41, range 40–59)	23.4 (SD 4.83, range 8.7–34.1)	27.82 (SD 5.39, range 11.9–41.6)

Between males and females, no significant differences were found in the neck–shaft angle (single hip: *p* = 0.88, pooled: *p* = 0.6), and the neck–trochanter angle in the transverse plane (single hip: *p* = 0.45; pooled: *p* = 0.1). There was a weak, but statistically significant correlation of age and neck/shaft angle (*R* = − 0.20, *p* < 0.05). There were no statistically significant correlations between age and any of the evaluated parameters, including neck/shaft angle, (*p* > 0.05).

The position of the fovea in the semi-coronal plane (true anteroposterior view) was always inferior to the longitudinal axis of the femur, what corresponds to the lower half of the femoral head. The fovea was positioned slightly more superior in women (mean difference in fovea angle in single hip: 5.7, *p* < 0.001, in pooled data: 5.850, *p* < 0.001) than men, as indicated by the fovea angle shown in Table 1. There was a weak but statistically significant correlation between the neck–shaft angle and the fovea position in pooled data (*R* = − 0.21, *p* < 0.05), indicating a tendency to more superior position with increasing valgus. This tendency did not show in single hip data (*R* = − 0.17, *p* > 0.05).

**Fig. 5:**
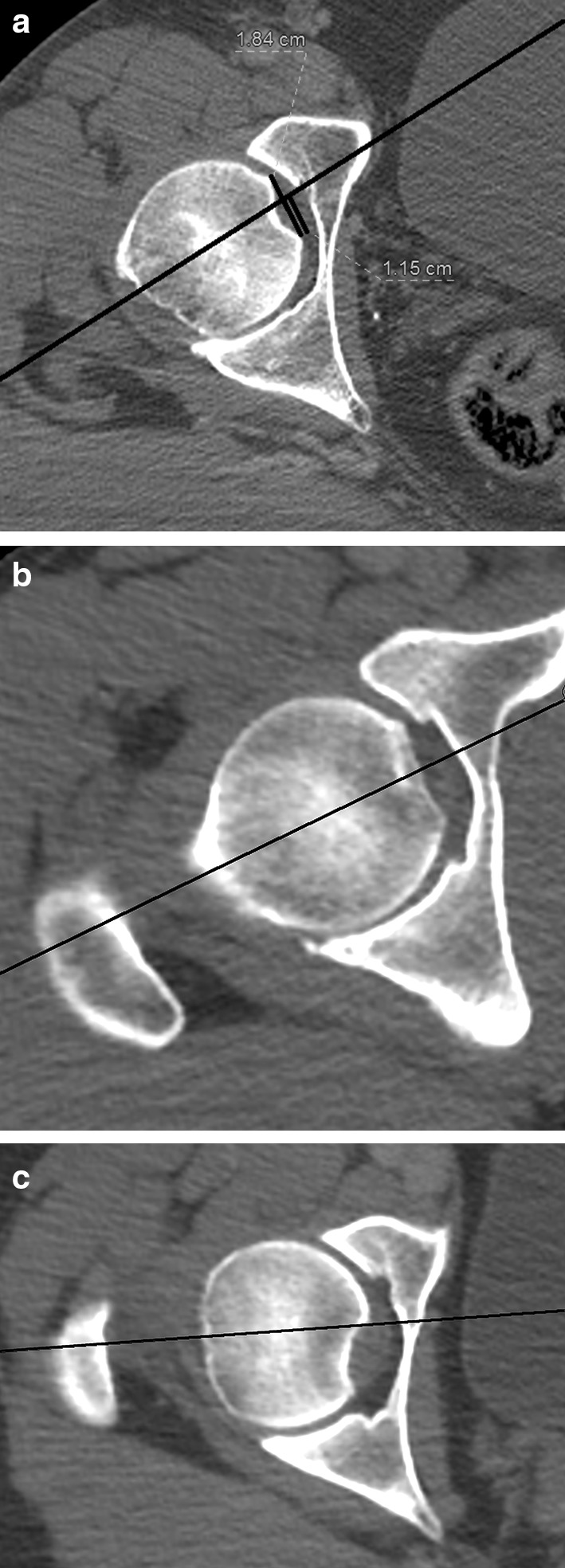
Location of the fovea in relation with the femoral neck axis in the transverse view. **a** Posterocentral. **b** Central. **c** Posterior

In the transverse plane, the axis crossed the fovea in every case in the anterior half of the fovea; therefore, always, half or more of the fovea diameter was posterior to the axis (Fig. 5a). As shown in Table 1, approximately ¾ of the fovea diameter in the transverse plane is located posterior to the neck axis. This number is always between 50 and 100%. However, the axis never passed outside the fovea. Only in some cases, the axis was directly central in the fovea or at the anterior border (Fig. 5b, c).

There were no statistically significant differences between the left and right hips in the parameters, as shown in Tables 1 and 2 (*p* > 0.05). Some slight differences were found between men and women. Men had significantly larger femoral heads diameter (approximately 5 mm in pooled data, 6 mm in single hip data, *p* < 0.001). In women, the fovea is slightly more posterior than in men, with on average 5% more of the fovea diameter in transverse plane is located posterior to the neck axis, but this tendency was significant only in pooled data (*p* = 0.02), and not in single hip data (*p* = 0.07). The transverse diameter of the fovea was larger in men, with average differences of 0.97 mm (*p* = 0.006) in pooled data, but this difference was insignificant when only single hip was evaluated (0.99 mm, *p* = 0.052). Slightly smaller average difference in size was found in the semi-coronal diameter (single hip: 0.56 mm, pooled: 0.67 mm), but statistically insignificant (*p* = 0.25 and *p* = 0.48, respectively).

The size of the fovea was significantly correlated with the size of the femoral head in both in the semi-coronal plane (*R* = 0.37, *p* < 0.05 in both single hip and pooled data) and in the transverse plane (single hip: *R* = 0.4, *p* < 0.05; pooled data: *R* = 0.38, *p* < 0.05).

The Acar’s index in women was slightly larger in both planes in women, but statistical analysis of single hip data and pooled data contradicts each other. In pooled data, in the semi-coronal plane, it was larger in women on average 1.58% (*p* = 0.03) and in the transverse plane 1.78% (*p* = 0.001). In single hip data, in the semi-coronal plane, it was larger in women on average 1.87% (*p* = 0.04) and in the transverse plane 1.66% (*p* = 0.11). This shows that the fovea probably covers a slightly larger area of the femoral head in women than in men.

## Discussion

The fovea and femoral head ligament has been more intensively studied in recent years. It is present in every hip, despite the fact that the femoral head ligament that is attached to it may be missing [17]. The function of the ligament itself is not well understood. It seems to serve as a stabilizer of the hip joint, and some authors recommend its preservation and using as an additional stabilizer in pediatric hip surgery [13]. There is some controversy around the importance of the blood vessels in the ligament, which plays a role in the fetal period, and then becomes increasingly less important with advancing age [1], but the vascular foramina are still present in 76% of adult femora [18].

The slightly posterior position of the fovea is mentioned in several studies, but as an introductory information cited from other sources or as a generally known fact, not as an investigated factor [2–6]. This study provides detailed information on the position of the fovea in relation with the femoral neck axis in two planes and its relationship to standard morphological parameters of the femur. The fovea has not been investigated this way before [8]; therefore, the parameters detailed in this study may aid future research in the anatomy, physiology, and pathology of the hip joint in general and the ligamentum capitis femoris in particular.

Several studies were performed to link the position of the fovea in the AP view with developmental dysplasia of the hip. It was shown that the fovea is located more superiorly in dysplastic hips, mostly because of increased femoral valgus found in this condition [4, 10]. This study is in concordance with this finding, as it shows that the fovea is more superior in more valgus hips, even in cases within normal range of the femoral neck–shaft angle; however, the statistical correlation in our study is weak (*R* = − 0.21), and found only in pooled data, but not in single hip data (*R* = − 0.17, *p* > 0.05). Moreover, the fovea was found to be more superior in women than in men despite the fact that the neck–shaft angle differences are insignificant and only 0.3100 greater on average in women (Table 1), what logically contradicts any strong relationship between the angle and fovea position.

Our study shows a weak but significant correlation of age and neck/shaft angle, indicating in concordance with other studies that femoral neck valgus decreases with age [19, 20]. However, no other parameter showed any correlation with age. Evaluation of age-related changes can be done only in a different study with a broader population, since this study is relatively smaller than other studies that center on age-related changes [19, 20].

It should be noted that the position of the fovea was assessed in relation with the neck axis, and not the acetabular sourcil, as in the standard method of delta angle measurement [4, 9, 10]; therefore, our findings cannot be directly related to the previous ones. Moreover, the delta angle, as it evaluates the spatial relations between two mobile elements, can be influenced by different adductions/abductions of the hip joint during image acquisition. A different approach to measurement was chosen in this study to provide a constant reference point, as well as because its primary interest was the femur, and not its relationship to the acetabulum.

A similar method of using the femoral neck axis as a reference point was used to assess the position of the fovea in transverse view. A delta angle in the transverse plane would be unreliable, because patients usually do not have CT scans performed with a standardized rotation, as would be needed to provide reproducible results [2]. In this case, the axis passed always within the fovea, what allowed providing an information that would be easy to visualize, that is the percentage of the fovea diameter that is posterior vs anterior to the femoral neck axis. Moreover, an angle between the axis and the edge of the fovea is difficult to comprehend and could be easily mistaken with the method that was used for the assessment in the semi-coronal view, where the angle was outside the fovea and in the transverse view would need to be inside the fovea.

This study shows, in concordance with others [3, 5, 7, 8], that the fovea is always located slightly posteriorly on the femoral head. Its position seems to be unrelated to femoral neck version. The method of neck–trochanter angle was used, as full femur scans were rarely available. It was shown that there is a stable relationship between the lesser trochanter version and the posterior condylar line, providing a reliable method of assessing femoral neck version in cases, where the femoral condyles are unavailable [16, 18]. Surprisingly, the neck–trochanter angle values were not correlated with the position of the fovea in the transverse view (*R* = 0.046, *p* > 0.05). In this study, the neck–trochanter angle was different from in the literature, with a much greater range of values [16]; therefore, both the reliability of the version assessment using the neck–trochanter angle and its relation to fovea position should be confirmed in further studies.

The size of the fovea, as estimated by its diameters, was slightly greater in the transverse plane than in the semi-coronal plane, contrary to the findings of others [8]. This may be attributed to differences in the studied group and methodology (cadaver vs CT scans), but requires further investigation in another study. The fovea diameter is greater in men than women, as its size is proportional to the size of the femoral head. It is, however, proportionally bigger in women when related to the size of the femoral head, as shown in the Acar’s index [2]. These differences cannot be attributed to age or proximal femoral geometry, because these parameters were similar in both groups.

This study was designed to assess just the position of the fovea on CT scans. The ligament of the head of the femur, which attaches to the fovea, was not evaluated, since it would require an MRI, which was not available. It should be noted, however, that the condition of the ligament may perhaps influence the size and shape of the fovea. If such is the case, its radiologic appearance may provide some indirect information on possible ligament lesions. In this study, we provided a reliable methodology of fovea assessment in the CT scans, what can be used in other, more advanced studies. Ideally, a combined MRI/CT/arthrography/arthroscopy study would allow a full assessment of the ligamentum capitis femoris and its bony insertions [12, 21].

This study is smaller than other population studies and some small gender, age and side differences may not reach its statistical significance. It has been designed to study only the anatomy and care was taken to avoid any bias related to hip pathology. As mentioned before, the importance of the position and morphology of the fovea in CT regarding hip pathology have yet to be proven, in other study. The methodology used in this study probably requires some refinement and more rigorous assessment of its reliability.

Our study gives an easy to remember information that the femoral neck axis never passes through the posterior half of the fovea, and confirms the opinions and findings of others [4, 8]. A radiographic study of the anteroposterior position of the fovea has not been conducted before. Providing a stable radiographic reference point in transverse scans in normal hips may help in assessing pathological conditions, as was described for anteroposterior radiograph of the hip joint in hip dysplasia.
